# Effects of Quinacrine on Expression of Hippo signaling Pathway Components (*LATS1, LATS2, *and *YAP*) in Human Breast Cancer Stem Cells

**DOI:** 10.31557/APJCP.2020.21.11.3171

**Published:** 2020-11

**Authors:** Soroush Darbankhales, Reza Mirfakhraie, Hossein Ghahremani, Mohsen Asadolahi, Kobra Saket-Kisomi, Lily Safakish, Sepideh Darbeheshti, Zahra Ganjkhanlou, Siamak Salami, Majid Sirati-Sabet

**Affiliations:** 1 *Department of Clinical Biochemistry, School of Medicine, Shahid Beheshti University of Medical Sciences, Tehran, Iran. *; 2 *Department of Medical Genetic, School of Medicine, Shahid Beheshti University of Medical Sciences, Tehran, Iran. *

**Keywords:** Cancer stem cells, Hippo signaling pathway, MDA-MB 231, quinacrine, triple negative breast cancer

## Abstract

**Objective:**

The Hippo signaling pathway has important role in the pathogenesis of some tumors. Breast cancer is the most prevalent cancer among females in the world. In recent years, various articles referred to inhibiting effect of quinacrine, a derivative of 9-aminoacridine, on the growth of several types of cancer cells. In this study, we evaluated the effect of quinacrine on expression of *LATS1, LATS2*, and *YAP* genes of the Hippo signaling pathway and *YAP* level in human breast cancer stem cells (MDA-MB 231 cell line). This cell line of breast cancer expresses the triple negative characteristics.

**Methods:**

MDA-MB 231 cells was treated with 0.5 µM of quinacrine for 3 days. The dose was selected using MTT assays. The expression of genes was quantified by Real-time PCR. The protein expression was performed by Western blotting. Significance of observations were checked by means of Mann-Whitney test using p<0.05 as the level of significance.

**Results:**

The present study demonstrated that expression of *YAP* gene in MDA-MB 231 cell line significantly down regulated by quinacrine. Quinacrine significantly decreased the amount of YAP protein. Moreover, quinacrine did not have meaningful effect on *LATS1* and *LATS2* genes expression.

**Conclusion:**

*YAP* gene is an oncogene and inhibition of its expression by quinacrine could effect on breast cancer cell progression.

## Introduction

Signaling pathways have an important role for communication between cells in an organism (Zhang et al., 2020). Some signaling pathways control the tissue development (Dreesen and Brivanlou, 2007). The Hippo signaling pathway is one of the pathways that can play an important role in the survival of cancer cells, with control of proliferation and apoptosis (Yu et al., 2015; Azad et al., 2019). Some important tumor suppressor genes (*LATS1 *and *LATS2*) and an oncogene (*YAP*) exist in this pathway (Shi et al., 2015). Deregulation of the Hippo signaling pathway has a significant effect on growth of some cancers (Yu et al., 2015). It is shown that some members of the Hippo signaling pathway is important in initiation and/or progression in human breast cancer (Cordenonsi et al., 2011; Guo et al., 2019; Kashihara et al., 2019). When the Hippo signaling pathway does not stimulate, some transcription factors such as TEAD family transcription factors bind to YAP (yes-associated protein) in nucleus. These transcription factors increase the expression of some genes that play an important role in cell proliferation, apoptosis and metabolism (Yu and Guan, 2013; Kashihara and Sadoshima, 2019). Therefore, the use of substances to down-regulate the expression of the *YAP* oncogene in the Hippo signaling pathway is important in inhibition of cancer development (Zhou et al., 2016).

Cancer can result from abnormal growth of cells which tend to proliferate in an uncontrolled way (Torre et al., 2015). Breast cancer, which mostly appears in postmenopausal women, is the most prevalent invasive malignancy in women. About 15 to 25 percent of breast cancer cells are missing estrogen and progesterone receptors and Her2 protein and refers to triple negative breast cancer (TNBC). This type of breast cancer cells is typically resistance to chemotherapy. TNBC is more common in younger patients and expresses the stem cells characteristics (Chavez et al., 2010; Alipour et al., 2013). 

Quinacrine (known as mepacrine, Atabrine, or Atebrin), a derivative of 9-aminoacridine, influences tumor cell proliferation by inhibition of signal transduction pathways (Guo et al., 2009) and autophagy (Khurana et al., 2015). Pharmacological properties of quinacrine discovered in 1912. This drug was used for antimalarial properties during World War II. Quinacrine has anti-inflammatory and antiautoimmune activities, too (Ehsanian et al., 2011).

The purpose of this study was to investigate the effect of quinacrine on expression of *LATS1, LATS2*, and* YAP* genes of the Hippo signaling pathway and *YAP* level in MDA-MB 231 cell line. MDA-MB 231 cells are the triple negative breast cancer cell line and express the stem cell characteristics (Chavez et al., 2010).

## Materials and Methods

*Cell culture*

The human MDA-MB 231 cell line was obtained from National Cell Bank of Iran (NCBI). The cells were cultured in DMEM and supplemented with fetal bovine serum (10%) and antibiotics (1% Penicillin-Streptomycin) at 5% CO_2_ and at 37°C.

*MTT assay*

Cytotoxic effect of quinacrine was evaluated by 3-(4,5-dimethylthiazol-2-yl)-2,5-diphenyltetrazolium (MTT) assay. Quinacrine was purchased from Sigma (St. Louis, USA). Stock solution of quinacrine was prepared in DMEM and stored at 4°C. About 5,000 cells were seeded in 96-well tissue-culture plates and grown for 24 hour and then treated with 0.5, 1, 2, 5, 10, 15, 20, 50, 100, and 200 µM of quinacrine. The control group was untreated cells. After 72 hour of treatment, 5 μL of MTT solution (5 mg/mL) was added to each well. Cells were incubated for 3 hour at 37°C. Then, 100 µl of dimethyl sulfoxide (DMSO) was added to each well after removing of medium. The color magnitude of wells was measured after 5 minutes of mild shaking by ELISA reader at 570 nm and reference filter of 630 nm. IC_50_ and IC_5_ were calculated by GraphPad Prism 6 software. 

*Real-time PCR*

After exposure to 0.5 µM quinacrine (72 hr), total RNA of cells was extracted from control and treated cells using the CinnaPureTM kit (CinnaGen, Tehran, Iran). The quality and quantity of the extracted RNA samples were evaluated by agarose gel electrophoresis and 260/280 absorption ratio of RNA preparations. Genomic DNA contamination from RNA samples was removed by DNase I kit that was purchased from ThermoFisher Scientific (Darmstadt, Germany). Synthesis of cDNA was carried out by cDNA synthesis kit as manufacturer’s protocol. RevertAid First Strand cDNA Synthesis Kit was obtained from ThermoFisher Scientific (Darmstadt, Germany). The SYBR Green quantitative Real-time PCR was accomplished by Real-time PCR machine (Rotor Gene 6000, Corbett). Amplicon RealQ PCR master mix was used for PCR amplification. First, an initial step of 4 min denaturation at 95°C was done for PCR amplification. Then, the amplification conditions were three-step 40 cycles (95°C, 10 seconds; 60°C, 20 seconds; 72°C, 15 seconds) and melting curve analysis at the final. The specificity of the primers was investigated using agarose gel electrophoresis and melting curve analysis. Data normalization was carried out by the *GAPDH* gene expression in each sample. The relative expression of target genes was calculated by evaluation of ΔC_t_ (Pfaffl, 2001). Electrophoresis of the PCR products was performed by agarose gel. Primer sequences for the genes analyzed are listed in Table 1.

*Western blotting analysis*

Cell lysates were provided by lysing the cells in RIPA lysis buffer containing a protease inhibitor cocktail. Briefly, after treatment (72 hr and 0.5 μM quinacrine), the cell lysate were centrifuged at 14,000 rpm for 10 minutes at 4°C. Then, the protein concentration was measured by the Bradford assay. For blotting, SDS-PAGE was carried out on 15% gel by loading 50 μg of protein per lane and completed with transferring protein onto a nitrocellulose membrane. Blocking of nitrocellulose membrane was done using 5% bovine serum albumin. Then, membrane was incubated overnight at 4°C with primary antibodies (YAP and actin antibodies) were procured from R & D Systems (Wiesbaden, Germany). For internal control, actin antibody was used. After 3 washes with buffer, the membranes were incubated with mouse secondary antibody (HRP-conjugated) for 1 hr at room temperature and then developed with an enhanced chemiluminescence (ECL) method on x-ray film. The magnitude of protein bands were evaluated using the ImageJ software.

*Statistical analysis*

Real-time PCR data was presented using medain value. Experiments were carried out in triplicate. Statistical analyses were performed using GraphPad Prism and Mann-Whitney test was used for Real-time PCR data. Western blotting analysis were performed by t test. Each set of experimental data was significantly evaluated using P-value less than or equal to 0.05.

## Results

*Antiproliferative activity of quinacrine*

Viability of MDA-MB 231 cells was evaluated using MTT assay for confirming the anti-proliferative activity of quinacrine on cells. Cell viability decreased with increasing concentrations of quinacrine (0–200 μM) for 72 hr with IC_50_ value of 3.5 μM. The IC_5_ value was 0.5 μM. For subsequent experiments, we chose 0.5 µM concentration of quinacrine for determining the effect of quinacrine on *LATS1*,* LATS2*, and* YAP* genes expression and Western blotting of* YAP*.

*Real-time PCR analysis*

**Figure 1:**
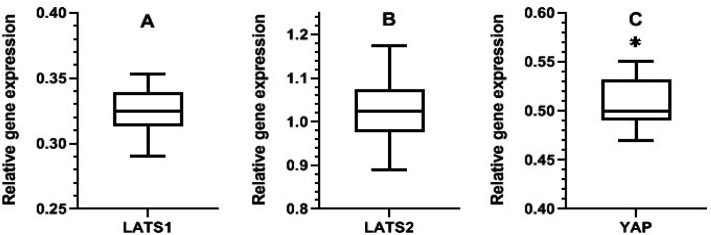
Alterations in the *LATS1, LATS2* and *YAP* Genes Expression in MDA-MB 231 Cells Treated with 0.5 μM Quinacrine for 3 Days (Box-Whisker Plot). Graphs represent median for at least three replicates and the significance of differences were defined by mann whitney test (P < 0.05). Significant down regulation of *YAP *expression (fold: −2.00 and P = 0.014) is evident in treated cells but not change the expression of *LATS1* and *LATS2* is evident in treated cells (* P<0.05).

**Figure 2:**
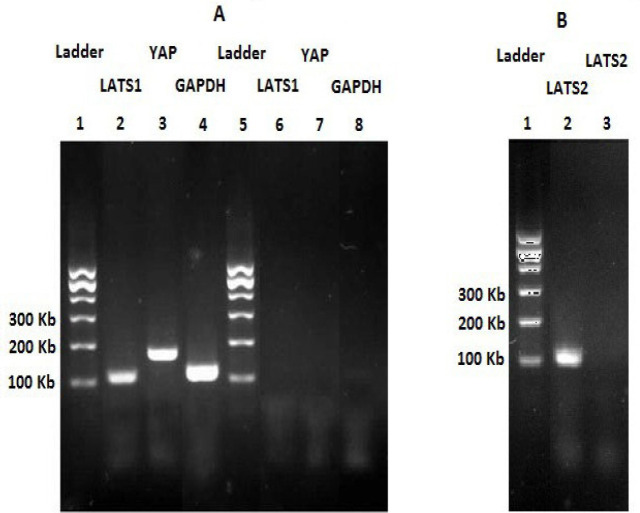
Agarose Gel Electrophoreses of PCR Products. A, PCR products of *LATS1, YAP,* and *GAPDH *genes (lines 6, 7, and 8 are negative controls), and B, PCR product of *LAT2* gene (line 3 is negative control)

As Figure 1 shows, quinacrine (0.5 µM) significantly down regulates the expression of* YAP* gene with fold change equal to −2.0 (P = 0.014) in the 72 hours treated cells. Quinacrine did not significantly alter the expression of *LATS1* and *LATS2* genes in the treated cells (P = 0.170 and 0.810, respectively). Agarose gel electrophoresis of PCR products is shown in Figure 2.

*Western blotting analysis*

**Figure 3:**
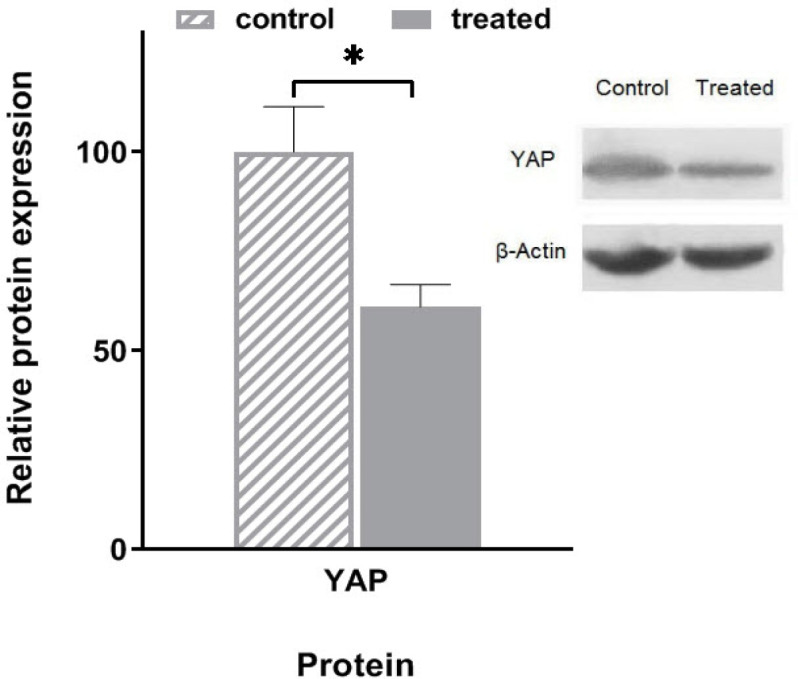
Expression Levels of YAP Protein. The expression of YAP oncoprotein was significantly less than control in MDA-MB 231 cells treated with 0.5 μM quinacrine for 3 days (fold: 61.0% ± 5.7 and P = 0.049). β-Actin was used as a housekeeping protein to normalize the expression (* P<0.05)

Analysis of YAP protein using Western blot significantly shows the amount of YAP protein decreased 39% in the 72 hours treated cells with quinacrine (Figure 3).

**Table 1:** Primer sequences of *LATS1, LATS2, YAP,* and *GAPDH* Genes

Gene	Amplicon size (bp)	Forward primer (5' to 3')	Reverse primer (5' to 3')
*LATS1*	110	CAAGATCCTCGACGAGAGCA	CCTTTCCAGCTCTGTTTGCG
*LATS2*	111	ACCCCAAAGTTCGGACCTTAT	CATTTGCCGGTTCACTTCTGC
*YAP*	170	TCCCAGCACAGCAAATTCTCC	AGGTGCCACTGTTAAGGAAAGG
*GAPDH*	116	GGTCTCCTCTGACTTCAACA	AGCCAAATTCGTTGTCATAC

## Discussion

Survival of cancer cells can be affected by inhibition of some cellular signaling pathways (Dreesen and Brivanlou, 2007). In the present study, we evaluated the effects of quinacrine on the Hippo signaling pathway of MDA-MB 231 cells. Our results showed that quinacrine significantly suppressed the expression of *YAP* gene of the Hippo signaling pathway. Moreover, YAP protein was down-regulated in MDA-MB 231 cells when treated with quinacrine. Breast cancer is the most commonly reported invasive malignancy in women with 450,000 deaths annually (Núñez et al., 2016). Breast cancer is more likely to have a triple negative pattern in the younger patients. TNBC cells have the characteristics of stem cells and have a bad prognosis (Alipour et al., 2013). It has been suggested that these cells are considered as separate populations in the tumor and cause recurrence of the disease after treatment (Al-Hajj et al., 2003). The characteristics of TNBC cells become visible in MDA-MB 231 cells (Chavez et al., 2010). Efforts to reprogramming and induce differentiation or apoptosis through manipulating of different message signaling pathways can be effective in improving the invasive behavior of such cells and facilitate the response them to treatment. 

Quinacrine is one of the most important derivatives of 9-aminoacridine. This drug was used as an antimalarial drug. This compound has also been shown to have anticancer activity (Ehsanian et al., 2011). Quinacrine can bind to DNA and effect on DNA function. It has been reported that in addition to the binding property of quinacrine to DNA, the anticancer activity of quinacrine can also related to the inhibition of signaling pathways in cancer cells. Quinacrine can affect survival signaling pathways in cancer cells such as Wnt/β-catenin and AKT pathways (Demeunynck et al., 2001; Guo et al., 2009). Quinacrine can reduce the invasive behavior of cancer cells by acting on the arachidonic acid pathway (Ehsanian et al., 2011). In addition, it has been shown that quinacrine can interrupt the growth process of some cancer cells by effect on some of the nuclear enzymes such as topoisomerase (Preet et al., 2012). Also, the effect of quinacrine on induction of cell death has been reported through increased expression of p53 protein in some cancers (Guo et al., 2009; Preet et al., 2012). It has been shown that the effect of quinacrine on the prevention of growth of breast cancer cells is much more severe than normal breast cells. In the study of Preet et al, the effect of quinacrine on the MDA-10A cell line was evaluated. This cell line have normal breast epithelial characteristics and it was observed that quinacrine, at a concentration of 200 μM, caused a 60% decrease in growth in these cells (Preet et al., 2012). We used a concentration of 0.5 μM quinacrine in our study, which is much less than the concentration studied in that study. This concentration is equal to IC_5_ value of quinacrine in the present study and at this concentration quinacrine has no toxic effect on the target cells.

The Hippo signaling pathway, in addition to controlling the growth and development of organs, plays an important role in the function of cancer stem cells (Yu et al., 2015). All organs are not equally sensitive to this signaling pathway. The most important components involved in this pathway are two protein kinases, *LATS1* and *LATS2*, and an oncogene called *YAP*. YAP interacts with TEAD family transcription factors as transcription coactivators. When the YAP protein is phosphorylated on the side chain of its serine residues by LATS1 and LATS2 proteins, its ability to interact with some of the cytoplasmic proteins are provided. Stabilization of YAP protein in the cytoplasm prevents its from entering the nucleus (Piccolo et al., 2014). In recent years, attention to YAP, as important protein of the Hippo signaling pathway, has increased for its remarkable deregulation in various human malignancies. YAP has corepressor and coactivator activity and for controlling the Hippo signaling pathway function is a significant target (Zhao et al., 2007; Zhao et al., 2008). Phosphorylation of YAP protein by upstream regulators inhibits its function. Phosphorylated YAP cannot enter the nucleus and modify the expression of its target genes essential for cell migration and proliferation (Hao et al., 2008). Increasing the expression of* YAP *gene is a negative prognosis of various cancers (Guo et al., 2019). YAP activity has been reported to increase the expression of some proteins, such as CyclinE and Diap1, which contribute to cell growth. CyclinE accelerates the cell cycle and Diap1 acts as an inhibitor of apoptosis. YAP inhibition is considered as a way to stop the growth of some cancer cells and to sensitize cells to apoptosis (Zhao et al., 2008). YAP overexpression have been reported in some type of breast cancer including Luminal or ER+ (estrogen receptor α positive) subtypes, HER2-enriched tumours, and in triple-negative breast cancer (Kim et al., 2014; Lehn et al., 2014; Min Kim et al., 2015). Kim et al. investigated YAP expression in the luminal A, luminal B, HER-2 rich, and triple negative breast cancer (TNBC). They found elevated expression level of YAP in HER-2 subtype of breast cancer. Cytoplasmic YAP expression negatively affected disease-free survival in HER-2 subtype. They suggested YAP expression could be a prognostic marker for breast cancer patients (Kim et al., 2014). Lehn et al. observed positive correlation between YAP1 expression and cell proliferation in the ER- (estrogen receptor α negative) subgroup of breast cancer. In the ER+ (estrogen receptor α positive) subgroup, YAP1 expression was inversely correlated to histological grade and cell proliferation (Lehn et al., 2014). It has been reported that the YAP expression in metaplastic carcinoma is higher than other subtypes of TNBC (Min Kim et al., 2015). Our study showed that the level of *YAP* gene expression and YAP protein amount was significantly down-regulated after 72 hour treatment with 0.5 µM quinacrine in breast cancer cell line MDA-MB 231. It is important to note that quinacrine in a concentration of about IC_5_ can inhibit YAP protein. This effect can help to inhibit malignant cells proliferation. It is shown that some transcription factors regulate YAP expression (Zhu et al., 2015). GABP an Ets transcription factor family, c-Jun, β-catenin/TCF4 complexes and cAMP response element-binding protein (CREB) can facilitate YAP transcription (Zhang and Zhou, 2019). Quinacrine inhibits Wnt-TCF signaling in breast cancer cells (Preet et al., 2013). In this regard, inhibition of the Wnt signaling pathway by quinacrine may down-regulate YAP expression. 

YAP activity is associated with resistance to treatment and recurrence of cancer. Cancer cells cultured with high activity of this protein are resistant to treatment with 5-fluorouracil and doxorubicin (Gallant et al., 2011; Yu and Guan, 2013). Tamoxifen is used to treat breast cancer cells that have estrogen receptors. Estrogen has been shown to activate YAP by stimulating the G protein-coupled estrogen receptor (GPER), which increases tumor growth and resistance to tamoxifen (Zhou et al., 2015). As a result, inhibition of YAP experssion not only targets tumor progression, it can also increase the sensitivity of cancer cells to chemotherapy. Therefore, quinacrine can inhibit growth and induce apoptosis in cancer cells in combination with some drugs used in chemotherapy (Zhang et al., 2012). We suggest that the combination therapy of quinacrine and tamoxifen be evaluated in tamoxifen-resistance cells of breast cancer. 

LATS1, in addition to acting in the Hippo signaling pathway as a tumor suppressor, has other function. This protein plays an important role in maintenance of ploidy and the G1 checkpoint. LATS1 has a role in setting the transition from G2 to M in cell cycle through a negative effect on CDK1 (Iida et al., 2004). LATS2 like LATS1 is a tumor suppressor in the Hippo signaling pathway. LATS2 has an important role in genomic stability (Hori et al., 2000). This protein down-regulate G1/S transition in cell cycle through a negative effect on cyclinE/CDK2 (Li et al., 2003). It has been shown that LATS is a tumor suppressor in human cancer cells (Visser and Yang, 2010), and reduced expression of* LATS1* or *LATS2 *can promote aggressive phenotype in human breast cancers (Visser and Yang, 2010). Quinacrine with concenteration of 0.5 µM did not changes on *LATS1* and *LATS2* gene expression in our study. One reason could be the use of low concentrations of quinacrine in this study. we used the quinacrine concenteration near IC_5_.

The present study demonstrated that *YAP* down-regulated after exposure to 0.5 µM of quinacrine for 3 days in breast cancer cell line MDA-MB 231. This gene of the Hippo signaling pathway is associated in cell proliferation and induction of epithelial to mesenchymal transition which intribute to metastasis (Serrao et al., 2018; Park et al., 2019). Thus, down-regulation of *YAP*, have this potentials to prevent malignant cells proliferation and metastasis in breast cancer stem cells. Moreover, quinacrine did not have meaningful effect on *LATS1* and *LATS2* gene expression. With these results, we can conclude that quinacrine has purposefully effect on some special genes in the Hippo signaling pathway.
